# Nanostructure and stability of calcitonin amyloids

**DOI:** 10.1074/jbc.M116.770271

**Published:** 2017-03-10

**Authors:** Federica Rigoldi, Pierangelo Metrangolo, Alberto Redaelli, Alfonso Gautieri

**Affiliations:** From the ‡ Dipartimento di Elettronica, Informazione e Bioingegneria, Politecnico di Milano, Piazza Leonardo da Vinci 32, 20133 Milano, Italy,; the § Dipartimento di Chimica, Materiali e Ingegneria Chimica “Giulio Natta,” Politecnico di Milano, Via L. Mancinelli 7, 20131 Milano, Italy, and; the ¶ VTT – Tecnical Research Centre of Finland, Biologinkuja 7, 02150 Espoo, Finland

**Keywords:** aggregation, amyloid, molecular dynamics, protein misfolding, protein-protein interaction

## Abstract

Calcitonin is a 32-amino acid thyroid hormone that can form amyloid fibrils. The structural basis of the fibril formation and stabilization is still debated and poorly understood. The reason is that NMR data strongly suggest antiparallel β-sheet calcitonin assembly, whereas modeling studies on the short DFNKF peptide (corresponding to the sequence from Asp^15^ to Phe^19^ of human calcitonin and reported as the minimal amyloidogenic module) show that it assembles with parallel β-sheets. In this work, we first predict the structure of human calcitonin through two complementary molecular dynamics (MD) methods, finding that human calcitonin forms an α-helix. We use extensive MD simulations to compare previously proposed calcitonin fibril structures. We find that two conformations, the parallel arrangement and one of the possible antiparallel structures (with Asp^15^ and Phe^19^ aligned), are highly stable and ordered. Nonetheless, fibrils with parallel molecules show bulky loops formed by residues 1 to 7 located on the same side, which could limit or prevent the formation of larger amyloids. We investigate fibrils formed by the DFNKF peptide by simulating different arrangements of this amyloidogenic core sequence. We show that DFNKF fibrils are highly stable when assembled in parallel β-sheets, whereas they quickly unfold in antiparallel conformation. Our results indicate that the DFNKF peptide represents only partially the full-length calcitonin behavior. Contrary to the full-length polypeptide, in fact, the DFNKF sequence is not stable in antiparallel conformation, suggesting that the residue flanking the amyloidogenic peptide contributes to the stabilization of the experimentally observed antiparallel β-sheet packing.

## Introduction

Amyloid fibrils are protein assemblies often associated to serious diseases such as Alzheimer's, Creutzfeldt-Jakob's, Parkinson's, and medullary thyroid carcinoma. They are formed by soluble proteins, which assemble to form insoluble assemblies that are resistant to degradation. However, amyloid fibrils also perform a number of physiological roles, such as providing the core of protective envelopes of fish and insect eggs, essential amphipathic materials of fungi and bacteria, and a key constituent of spider silk (1).

Despite amyloidogenic proteins do not share all the same sequences, it has been shown that they share common structural features, such as the prevalence of β-sheet folding, the presence of a hydrophobic core, and the hierarchical organization (2–4). Unfortunately, the insolubility and low crystallinity of amyloid fibrils hinder the determination of their structures using X-ray crystallography and NMR. Nevertheless, the mechanisms of formation and the structure of amyloid fibrils are starting to be elucidated (5, 6), also thanks to the contribution of molecular dynamics (MD)^3^ simulations (7–9), which allows investigating the detail of molecular interactions responsible for fibril initiation and stabilization.

**Figure 1.**
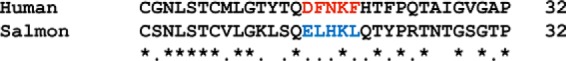
**Calcitonin sequence alignment.** Human and salmon calcitonins are aligned using ClustalW 1.82 (38). *Asterisks* denote identity, *dots* indicate similarity, and *no marks* denote very different residues. Despite the high homology (50% identity, 87.5% similarity) the salmon calcitonin is much less amyloidogenic. In particular, the amyloid-forming sequence in hCT (*red*) is rather different in the salmon calcitonin (*blue*).

Human calcitonin (hCT) is a thyroid hormone consisting of 32 amino acids and involved in calcium-phosphorous metabolism. In concentrated aqueous solution hCT forms amyloid fibrils with a diameter of 8 nm, which are associated with medullary carcinoma of the thyroid (10). According to previous works by the Gazit and co-workers (11), there is strong evidence that the DFNKF peptide (corresponding to residues ranging from Asp^15^ to Phe^19^ of hCT) is the minimal amyloidogenic unit of calcitonin. Indeed, this peptide quickly self-assembles into highly ordered fibrils and its truncation or mutation reduce or eliminate fibrillation (12). The fact that DFNKF is strongly amyloidogenic (but its mutations are not) can explain the lower amyloidogenic properties of salmon calcitonin (sCT), the hormone used for the treatment of hypercalcemia or osteoporosis, which presents two leucine residues (Leu^16^ and Leu^19^) instead of Phe^16^ and Phe^19^ in hCT (Fig. 1) (13).

Despite the solid knowledge on the sequence responsible for fibril formation and on the effects of point mutations, the structural basis of the fibril formation and stabilization is still mostly unknown. In particular, NMR studies suggested that hCT forms antiparallel β-sheets (14, 15), whereas other studies based on structural and MD simulations of DFNKF peptides (16) or crystal structures of halogenated DFNKF peptides (17) proposed a fibril assembly based on parallel β-sheets. However, these pivotal computational studies, by considering structures made of a few peptides, do not allow investigating the lateral interactions that may contribute to hold together several strands in a fully formed amyloid fibril.

In this work, we first aim to investigate the conformation of soluble hCT, for which the experimental structure is lacking. Second, we modeled for the first time amyloid fibrils with size comparable with experiments (up to 8 nm in diameter and 15 nm in length) formed either by full-length hCT and, for comparison, by DFNKF peptides. These fibril models allow us to investigate the stability of different proposed assemblies of full-length calcitonin and DFNKF peptides, so to disclose the likely structure of calcitonin amyloid fibrils.

## Results

### Structure of human calcitonin in diluted solutions

**Figure 2.**
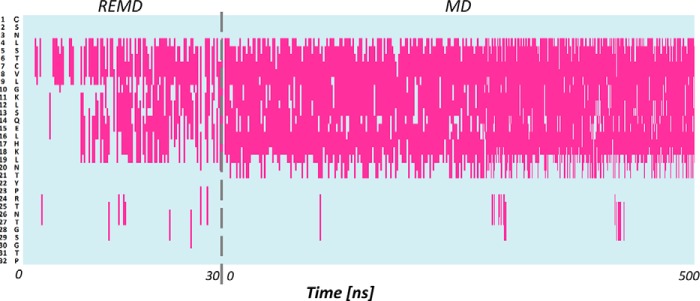
**Secondary structure of salmon calcitonin during REMD and MD simulations.** Salmon calcitonin amino acids are listed along the *y* axis and their secondary structure time evolution is represented along the *x* axis. *Pink* represents α helical conformation.

We provided the structure of hCT using two different and complementary approaches, as described under “Materials and methods.” In the first approach (*ab initio*), we build the extended structures of sCT and hCT and then perform 30-ns replica exchange molecular dynamics (REMD) simulations followed by 500-ns classical MD. We validate our protocol by comparing the structure of sCT predicted with our method with the one experimentally obtained with NMR (PDB code 2GLH). We first compare the r.m.s. deviation of our model using the NMR structure as a reference, during REMD simulation and during classical MD (see supplemental Fig. S1). The results show that r.m.s. deviation quickly decrease during the REMD simulation as calcitonin gradually forms an α-helix from residue 4 to 21, in agreement with the experimental structure. In the following MD simulation, the structure is highly stable, as shown by the small oscillation in r.m.s. deviation values. We compare the evolution of the helical content during REMD and MD simulations. The α-helix quickly develops during REMD and it stabilizes during the MD simulation (Fig. 2). In the last part of the MD simulation, the helix stably involves residues 5–19 and it often extends from residue 4 to 21, in high agreement with the experimental structure. It is important to note that the NMR structure is composed of 100 different conformations, which differ for the extension of the α-helix (variably terminating between residues 20 to 22) and for the arrangement of the disposition of coil termini. Overall, our results show a good agreement between the predicted and the experimental sCT structure (Fig. 4*A*), which is made of a long α-helix (residue 4–21) and a random coil C terminus.

**Figure 3.**
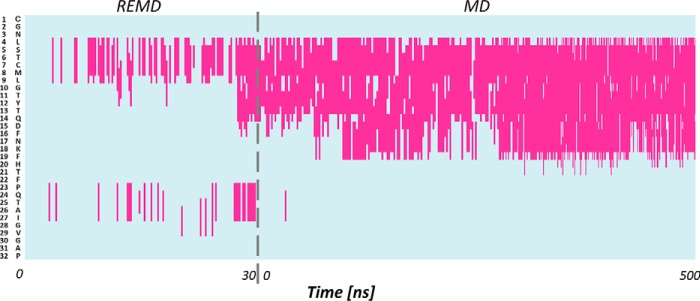
**Secondary structure of human calcitonin during REMD and MD simulations.** Human calcitonin amino acids are listed along the *y* axis and their secondary structure time evolution is represented along the *x* axis. *Pink* represents α helical conformation.

**Figure 4.**
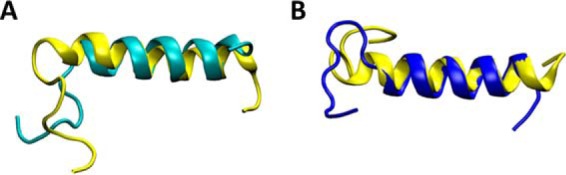
**Structural alignment of final MD models and NMR structure.** In *yellow,* the PDB code 2GLH experimental structure of sCT; in *cyan*, the *ab initio* predicted structure of sCT, at the end of the 500-ns MD simulations (*panel A*), showing excellent agreement. *Panel B* shows the predicted hCT structure at the end of a 500-ns long MD simulation (*blue*) that exhibits an extended helix ranging from residue 5 to 19. *Yellow* represents the experimental sCT structure for comparison.

The same protocol coupling REMD and classical MD is then applied to predict the structure of hCT. To assess the convergence of the simulations we monitor the polypeptide r.m.s. deviation using the sCT NMR structure as a reference. The results show that r.m.s. deviation quickly decreases during the REMD simulation as hCT gradually forms an α-helix and then stabilized during the MD simulation. Starting from the extended conformation, the REMD simulation results in an extended α-helix covering residues 5–14. In the following, 500-ns long classical MD simulation in explicit solvent the α-helix became more continuous including all residues in the range 4 to 19 (Fig. 3). The secondary structure timeline shows that despite the difference in primary sequence between, the structure adopted by hCT is very similar to that of sCT (r.m.s. deviation = 3.3 ± 0.18 Å) with α-helix as dominant motif for the secondary structure and covering residues 4–19 (Fig. 4*B*).

As a further validation of the predicted hCT structure, we used a homology modeling approach based on the sCT structure. In the initial homology model the helix covers residues 4–21 (as in the PBD code 2GLH template), whereas after MD the helix stabilize in the 5–19 range, the same range obtained with the *ab initio* protocol. The r.m.s. deviation difference in the helical section between the two predicted hCT structures is 0.47 ± 0.37 Å, which further confirms that the two independent methods yield the same hCT structure. The PDB structure of the final hCT model is available in the supplemental figures.

### Human calcitonin amyloid conformation

We investigated the likelihood of three previously proposed conformations of calcitonin amyloid fibrils. We first considered calcitonin octamers to assess the stability of fibril nucleation sites and subsequently we tested protofibrils made of 32 calcitonin molecules to test the influence of lateral interactions.

**Figure 5.**
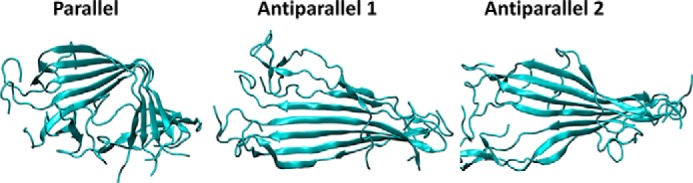
**Conformations of the different hCT octamers.** The stable structure of the three putative arrangements of hCT amyloids after 200-ns long classical MD simulation at 300 K.

All three octamer models reach structural equilibrium within 100 ns of simulation, thus we use the remaining 100 ns of simulation as production run to average quantitative results. In particular, the r.m.s. deviation is highly stable for all three conformations with fluctuations of less than 1 Å (see supplemental Fig. S2). The stable structures of hCT octamers are shown in Fig. 5. We observe that the parallel octamer forms a clear bend in the middle of the polypeptide chain, which could prevent the lateral aggregation of further calcitonin molecules. On the other hand, in both of the antiparallel octamers the polypeptide molecules maintain a straight conformation and assume a super-helical twist, which is compatible with experimental observation of helical fibrils.

**Figure 6.**
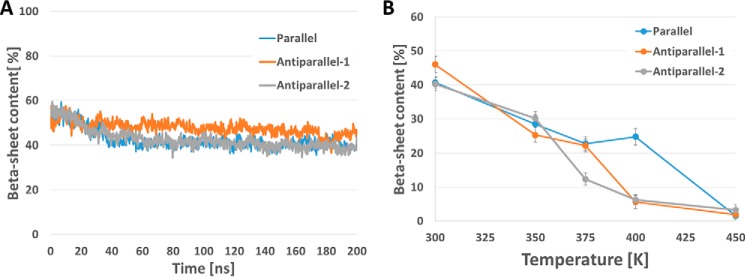
**β-Sheet in hCT octamers during MD simulations.** The β-sheet motif content for each octamer model is represented during the 200-ns MD simulations (*A*). The trends for the two antiparallel conformations are highly comparable, although the β-sheet content is slightly higher in the parallel arrangement. The thermal stability differs for the three models (*B*), showing that at 400 K only the parallel hCT octamers retains a significant content of β-sheets. The β-sheet content is the average after it reaches a stable value during the MD simulations at the different temperatures (300, 350, 375, 400, and 450 K).

For all three octamers, we analyzed the content of the β-sheet secondary motif during MD simulations (Fig. 6*A*). The β-sheet content stabilizes to 40.5 ± 1.8% for the parallel octamer, to 46.0 ± 2.4% for the antiparallel 1 model, and to 40.0 ± 1.9 for the antiparallel 2 model. The antiparallel 1 model thus presents the highest content of amino acid forming β-sheets, suggesting that this conformation is slightly more stable than the two other proposed models, in accordance with previous NMR results (13). To test the stability of each system we performed 100-ns long simulations at increasing temperatures (from 350 to 450 K), showing a decrease of the β-sheet content for all models (Fig. 6*B*). The results show that the β-sheet content decreases with the temperature for all systems, however, there is a clear drop at specific temperatures, which are different depending on the models, suggesting that the stability follows the order parallel > antiparallel 1 > antiparallel 2.

In addition to the octamers (representing nucleation sites), we investigate the stability of small hCT amyloid fibrils (protofibrils), constituted of four hCT octamers (*i.e.* 32 hCT molecules). All three systems reach r.m.s. deviation convergence within 80 ns of MD. The results show that antiparallel 1 arrangement presents a larger content of residues in the β-sheet conformation. In particular, during the last 20 ns of MD simulations, the β-sheet content is 24.45 ± 0.74% for the parallel arrangement, 30.5 ± 0.78% for antiparallel 1, and 21.7 ± 0.75% for antiparallel 2 model.

**Figure 7.**
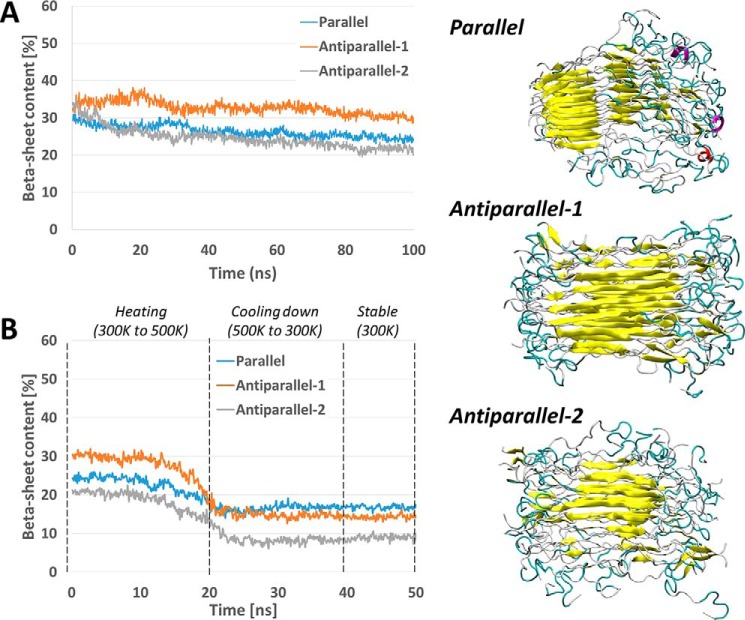
**β-Sheet content and structures of the different hCT protofibrils.** The variation of β-sheet content during the 100-ns MD simulation at 300 K (*A*) and during the Simulated Annealing MD simulation (*B*), showing that antiparallel 1 conformation has the highest degree of β-sheet organization, whereas the parallel arrangement is the most resistant to thermal stress. The stable structures of the three protofibrils are shown on the *right*.

The stability and the β-sheet content in the protofibrils is investigated also with Simulated Annealing, in which the models are rapidly heated to 500 K and cooled down to 300 K. In all models the β-sheet content rapidly decreases during the heating phase and remains stable during the cooling-down phase. The lost secondary structure motifs are not recovered during the third phase of the simulation (stable at 300 K for 100 ns). However, the antiparallel 1 and parallel conformations keep a comparable β-sheet content of ≈20%, thus confirming these two structures as possible calcitonin amyloid conformations, whereas the antiparallel 2 show a lower degree of ordered secondary structures (Fig. 7). We note that whereas the β-sheets involve the central part of the antiparallel 1 protofibril, in the parallel conformation the β-sheet content is located mostly in the C-terminal part of the molecules. This behavior is due to the bulky loop located in the N-terminal region and formed by residues 1–7 (linked by the Cys^1^-Cys^7^ disulfide bridge) that in the parallel model are located on the same side. This disordered region, although is not destabilizing in the protofibril models considered here, could limit the assembly and/or growth of larger hCT fibril.

### DFNKF amyloid peptides

**Figure 8.**
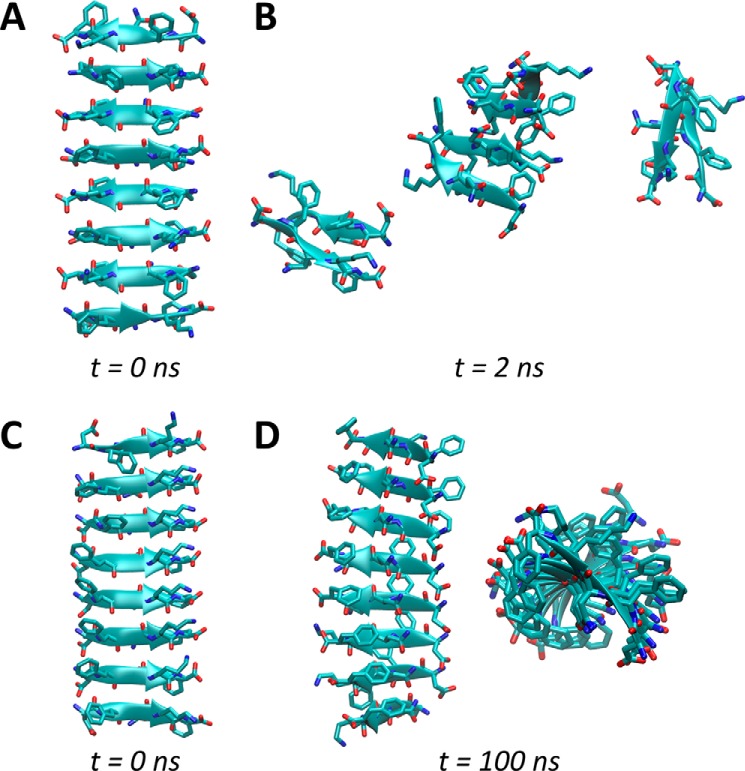
**Antiparallel and parallel conformations.** The antiparallel conformation (*A*) quickly results in the disruption of the octamer (*B*). The parallel arrangement of β-sheet (*C*) leads instead to the formation of a stable supramolecular helix that lasts for the whole MD simulation (*D*). The parallel conformation is stabilized by inter-strand hydrogen bonds (also found in the antiparallel conformation) and by aromatic interactions (π-π stacking) involving phenylalanine residues in positions 2 and 5. These hydrophobic interactions are not observed in the antiparallel arrangement.

To assess possible differences in the amyloids formed by full-length hCT and the minimal fibril forming peptide DFNKF, we investigate the stability of DFNKF octamers in parallel and in antiparallel conformations. For this purpose the peptides are stacked and then simulated for 100 ns in explicit water. The results clearly show that the parallel arrangement is extremely stable, as it forms a supramolecular helix that is retained for the whole simulated time. Conversely, the octamer in antiparallel arrangement quickly breaks apart in a few nanoseconds (Fig. 8). Both arrangements allow for the formation of inter-strand hydrogen bonds that stabilize the structures. However, only the parallel conformation allows for the occurrence of aromatic ring stacking of phenylalanine residues in positions 2 and 5, which significantly contribute to the overall stability of the fibril.

**Figure 9.**
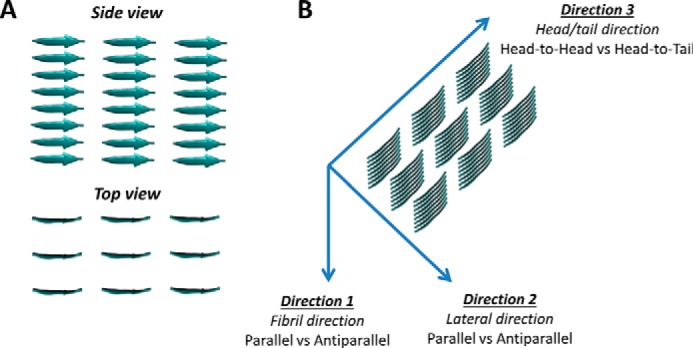
**Molecular models of DFNKF protofibrils.** Protofibrils made of 8 × 3 × 3 pentapeptides (*A*) are assembled combining all possible configurations in the three directions, leading to 8 different protofibril models (*B*). The naming of different protofibrils is Direction 1-Direction 2-Direction 3 (where *A,* antiparallel; *P*, parallel; *HT*, head-to-tail; *HH*, head-to-head). For example, “A-A-HH” is the protofibril with antiparallel configuration in Direction 1, antiparallel configuration in Direction 2, and head-to-head configuration in Direction 3.

It has been previously suggested that β-sheets oligomers represent the nucleation core of the amyloid fibrils during their formation (12). Here we show that the oligomers are not stable in the antiparallel configuration, hence the nucleation and thus the formation of fibrils will be unlikely. Conversely, the oligomers with parallel peptides show large stability, thus suggesting a higher potential to be the nucleation core of DFNKF fibril. However, to rule out the possibility that lateral interactions stabilize the antiparallel β-sheets, we investigated 72-mer DFNKF protofibrils using all possible configurations in the three directions, leading to eight different protofibrils (Fig. 9).

**Figure 10.**
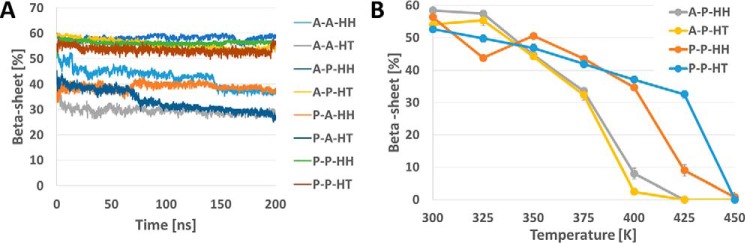
**β-Sheet content of DFNKF protofibrils.** The protofibrils with a parallel configuration in the lateral direction (*X*-P-*X*) show higher stability compared with all the other as observed during 100-ns simulations at room temperature (*A*). Simulations at increasing temperature show that the protofibrils with a parallel configuration in the fibril direction (i.e. P-P-*X*) show higher stability compared with the antiparallel configuration in the same direction. We excluded from this analysis the protofibrils with lateral antiparallel arrangement in (*X*-A-*X*) because they are unstable already at room temperature (*B*). The β-sheet content is averaged over all 72 pentamer at each time point. We note that the secondary structure analysis does not include the two terminal residues of the pentamer, hence the maximum β-sheet content is 60%.

The results show that the stability (in terms of secondary structure content) at room temperature is higher for models that have a parallel arrangement in lateral direction (Direction 2), whereas the configuration in the other two directions has a limited effect in the stability (Fig. 10*A*). To further assess the stability of the systems, we simulated the four most stable systems (A-P-HT, A-P-HH, P-P-HT, and P-P-HH) at 7 different fixed temperatures ranging from 300 to 450 K for 100 ns at each temperature.

The analysis of β-sheet content during simulations at increasing temperatures (Fig. 10*B*) shows that models with a parallel arrangement in Direction 1 (fibril axis) keep a higher content of secondary motifs during the heating process compared with antiparallel configuration in the same direction. These results show that the most stable protofibrils are those with parallel configuration in Direction 1 and Direction 2, with limited effect of the orientation in Direction 3 (*i.e.* P-P-HH and P-P-HT).

**Figure 11.**
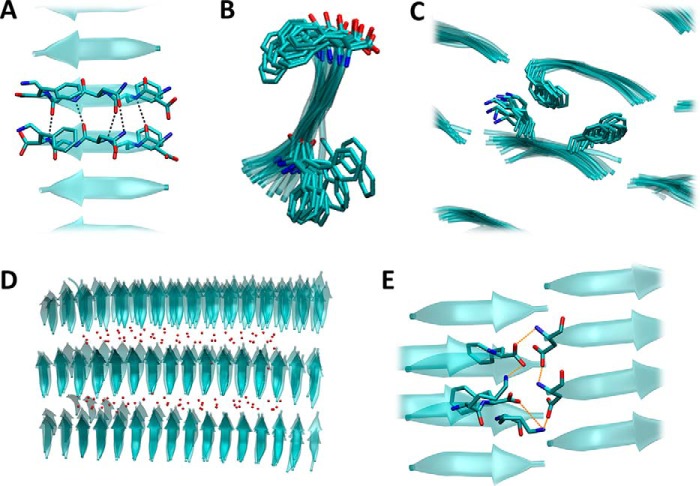
**Amyloid stabilizing interactions.** The DFNKF peptides are held together by intra-strand hydrogen bonds: four of these hydrogen bonds involve backbone-backbone interactions, where an additional hydrogen bond is observed between the side chains of stacked Asn^3^ residues (*A*). Further stabilization is provided by aromatic ring stacking of Phe residues (*B*). Lateral connections between DFNKF strands are due to hydrophobic interactions that involve, on one side, the aromatic ring of Phe^2^ and the alkane chain of Lys^4^ and, on the other side, the aromatic ring of Phe^5^ (*C*). The tight lateral interconnection between DFNKF strands defines the hydrophobic core of the amyloid fibril, from which the water molecules (*red dots*) are excluded (*D*). The head-to-tail connection between the strands is provided by salt bridges (up to 5 different types) involving the N-terminal (+), the C-terminal (−), Lys^4^ side (+), and Asp^1^ side chain (−) (*E*). This highly hydrophilic region is also where the totality of water molecules are located within the fibril.

The extremely stable and ordered structure of the protofibrils allows us a deeper insight into the different stabilizing molecular interactions. The in-depth analysis of interactions is performed on the P-P-HT protofibril, being the most stable. The peptides are connected by up to five highly stable hydrogen bonds (observed for >30% of simulated time) (Fig. 11*A*). The assembly is further stabilized by aromatic ring stacking of Phe^2^ and Phe^5^ residues (Fig. 11*B*). The two phenylalanine side chains, together with the alkane chain of Lys^4^, contribute to the formation of the tight hydrophobic core of the amyloid fibrils, which is responsible for the lateral inter-strand stabilization of the fibril (Fig. 11, *C* and *D*). Finally, a network of highly stable salt bridges (observed for >70% of simulated time) is responsible for the head-to-tail connectivity between strands (Fig. 11*E*).

## Discussion

We first investigated the structure of the calcitonin monomer because it is the building block of the calcitonin amyloids and, although it has been suggested that the monomer conformation has predominant α-helix content (similar to salmon calcitonin), its secondary structure has never been fully characterized by X-ray or NMR. Here, by modeling the single monomer (starting from an extended configuration) we show that the isolated monomer spontaneously folds as an α-helix. On the other hand, the presence of multiple monomers stabilize the extended conformation, leading to the formation of the amyloid fibril.

**Figure 12.**
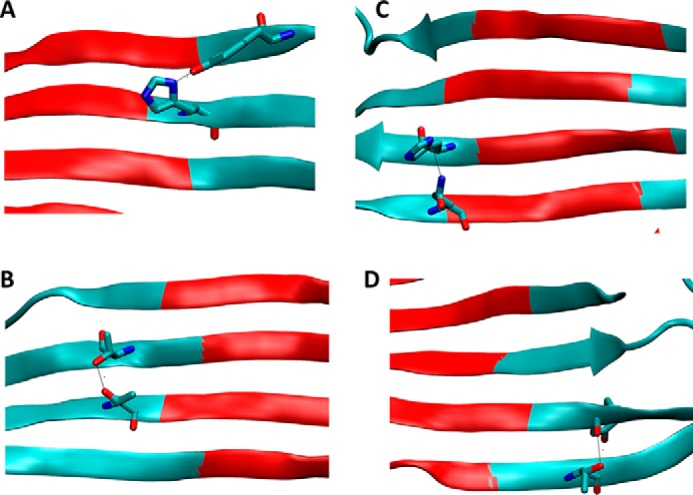
**Hydrogen bonds among calcitonin residues not included in DFNKF sequence.** *A–D*, four highly stable hydrogen bonds formed in the region excluded by DFNKF peptides (highlighted in *red*) may explain the ability of full-length hCT to assemble as antiparallel β-sheets, which is precluded to DFNKF alone.

A major open question concerning calcitonin fibrils involve the apparent discrepancy between NMR data on full-length hCT showing the prevalence of antiparallel β-sheets (14) and computational and structural studies of DFNKF peptides proposing a fibril assembly based on parallel β-sheets (16). Here we show that although DFNKF peptides are able to form stable fibrils only with parallel assembly, the full-length polypeptide forms amyloid structures both with parallel and antiparallel arrangements. The different stability of antiparallel conformations in DFNKF and hCT may be explained taking into account stabilizing interactions that involve residues flanking the DFNKF sequence in hCT. The analysis of hCT octamers and protofibrils in antiparallel 1 conformation highlights the presence of four highly stable (>50% of simulation time) intermolecular H-bonds that contribute to amyloid stabilization. These interactions involve residues Tyr^12^-His^20^, Thr^21^-Thr^13^, Gln^14^-His^20^, Thr^21^-Thr^12^ (Fig. 12). The lack of these interactions when only the DFNKF sequence is considered may explain the different behaviors between the pentapeptide and full-length calcitonin.

**Figure 13.**
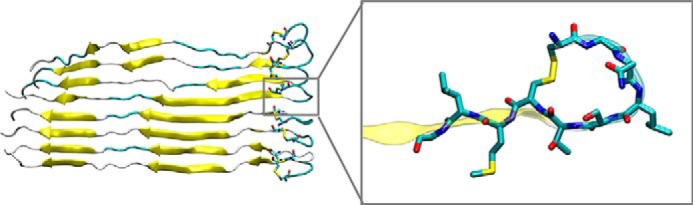
**N-terminal loops.** The figure shows the initial packing of a parallel octamer, highlighting how the bulky loops (*inset*) on the same side may destabilize the fibrils. The simulation of small protofibrils shows that, although the amyloid structure is stable, the β-sheets are located only in the C-terminal section, whereas the N-terminal region is highly disordered. This behavior could limit or prevent the formation of larger amyloid with parallel arrangement of hCT molecules.

On the other hand, we observe that full-length hCT present stable structures with parallel β-sheets, which are not observed in experiments. Nonetheless, despite the high stability, the models of the nucleation core (octamers) assembled with parallel β-sheets present a marked bend in the central part of the octamer, which could prevent or delay lateral aggregation. The protofibril models with parallel molecules show that β-sheets involve only the C-terminal section of the molecule because the bulky loops formed by residues 1 to 7 are located on the same side (Fig. 13). Although this phenomenon does not disrupt the protofibrils considered here, it can hinder the aggregation or extension of larger fibrils. Hence, although we cannot rule out the possibility of hCT forming parallel β-sheets, there is evidence for a preferential aggregation with the antiparallel conformation.

## Conclusions

### Diluted human calcitonin forms an α-helix

The first major finding of this work is the structure of the soluble hCT, which was previously unavailable, showing that it is made of a continuous helix ranging from residue 5 to 19, a structure very similar to that of sCT. The same hCT structure is obtained with two independent methods, *ab initio* folding based on REMD and homology modeling, showing the high reliability of the proposed structure.

### Human calcitonin forms amyloid fibrils in antiparallel conformation

We showed that full-length hCT arranges as amyloid fibril preferably as an antiparallel conformation with Asp^15^ and Phe^19^ aligned. This structure provide the highest content of β-sheets motif at room temperature and it is the conformation proposed in previous NMR studies, which reported that this conformation accounts for 70% of hCT amyloids (14). A further finding of this work is that hCT behaves differently from DFNKF peptides, which assemble only in parallel β-sheets. Our MD simulations show that antiparallel assembly quickly unfolds, whereas parallel β-sheets hold the structure for the whole 100-ns simulation. This finding confirms the results of previous work on DFNKF peptides (16), in which MD simulations up to 4 ns suggested a parallel assembly. The results shown here thus provides evidence that the full-length hCT and the DFNKF peptide may assemble in a different way, explaining the apparent discrepancy between NMR data and previous MD results.

The detailed knowledge of hCT assembly and the molecular model developed here may help to better understand and provide a solution against amyloid formation (18). For example, a recent study found evidence that molecules such as magnolol and honokiol have inhibitory effects of hCT aggregation (19). Our models can be used to investigate the detailed interactions of these molecules with hCT fibrils and provide the basis for further drug optimization. Similarly, the insights into DFNKF peptides assembly can be crucial for nanobiotechnological applications (20–22), thanks to the possibility to tune their properties, for example, by halogenation (23) or site-specific functionalization (24).

## Materials and methods

### Structure of human calcitonin in diluted solutions

We investigated the structure of soluble hCT with two complementary approaches. In the first method (*ab initio*), we started from the extended structure and used REMD simulation followed by extensive MD simulation. The protocol is validated by testing on sCT, for which the folded structure is known (Protein Data Bank code 2GLH). In the second method (homology modeling), we used the structure of sCT as a template to generate the initial folding of hCT, then the hCT model is extensively equilibrated. Finally, we compared the structures obtained with the two independent methods.

For the *ab initio* method we built the starting (extended) conformations of both sCT and hCT using PyMol (25). The disulfide bond between cysteines residues in positions 1 and 7 is included during topology building, then we minimize the structure while applying a position restrain (with a 1 kcal mol^−1^ Å^−2^ spring constant) to the backbone of residues from 12 to 32, to allow the relaxation of the disulfide bond. To obtain an accurate folded structure of hCT in solution we perform REMD (26) followed by extensive classic MD simulation in explicit solvent. We validated our protocol using sCT whose three-dimensional crystallized structure is available in the Protein Data Bank (PDB code 2GLH) (27). Starting from the initial extended model of both polypeptides, we ran 30-ns REMD simulations using NAMD code (28) and CHARMM22 force field with C-map correction (29) in a vacuum with a temperature range between 300 and 500 K. From the trajectories, we extracted the frames at 300 K and clusterized hCT and sCT conformations with the VMD clustering tool (with 1.0-Å cutoff and restricting analysis to amino acids from 4 to 23 to avoid taking in account floppy terminals). For both sCT and hCT we selected one frame from the most populated conformational cluster and solvated the polypeptide using the TIP3P explicit solvent model (30) and add ions to neutralize the system. The setup resulted in a ≈63,000 atom system in a simulation box of initial dimensions 95 × 87 × 80 Å^3^. The system was minimized and equilibrated using the NAMD code (28) under constant pressure and temperature (NPT) conditions to relax the volume of the periodic box. The pressure was set to 1 atm and the temperature to 300 K, whereas using a time step of 2 fs, a non-bonded cut-off of 9 Å, rigid bonds, and particle-mesh Ewald long-range electrostatics. Subsequently, we performed 500-ns long MD simulations at 300 K to stabilize the structure in explicit solvent. We employed ACEMD (31) on a NVIDIA Kepler K40 GPU using a time step of 4 fs, and a NVT ensemble. All other parameters (temperature, non-bonded cut-off, and PME) are the same as in the equilibration phase. We evaluated the stability of the systems by monitoring the convergence of the r.m.s. deviation in VMD (32) software.

To confirm the structure of hCT obtained with the *ab initio* method, we built a new structure using homology modeling based on the sCT structure. We used Modeler software (33), building 500 models and choosing the best result according to DOPE score. We then simulated the resulting polypeptide for 200 ns in explicit solvent using ACEMD software. All MD parameters were kept the same as discussed above and the structure was analyzed (after r.m.s. deviation convergence) in terms of secondary structure stability and extension.

### Human calcitonin amyloid conformation

To elucidate the structure of hCT amyloid we first investigated hCT octamers in three different conformations, because the formation of β-strands has been proposed as the first step in amyloid nucleation (12). On the basis of NMR studies, Naito and colleagues (13) proposed two antiparallel packing conformations that differ for displacement along the polypeptide axis. In addition to these two packing conformations, we tested the stability of the parallel arrangement, based on previous works (14), suggesting that DFNKF peptides assemble in parallel conformation (Table 1, models 1, 2, 3). The initial assembly in the form of parallel or antiparallel β-sheets is performed in VMD (32). The single monomer in extended configuration is aligned along the *x* axis, then we make 7 copies of the monomer and we displace the copies along the *y* axis by multiples of 5 Å. The distance is chosen to avoid atom clashing but at the same time allowing the formation of intermolecular interactions and thus avoiding the separation of the chains. A similar distance has been used in the past to model assembly of DFNKF peptides (16). In the case of antiparallel octamers, every second monomer is rotated by 180° around the *y* axis and displaced according to NMR studies (13) (see also Table 1). We then minimize the structure while applying a position restrain to the backbone of residues from 12 to 32, to allow the relaxation of the disulfide bond between Cys^1^ and Cys^7^. The systems were then solvated with TIP3P water molecules in a periodic box. The molecular models are minimized and equilibrated following protocols used in previous studies (34–36). Briefly, we use NAMD code (28) under constant pressure and temperature (NPT) conditions to relax the volume of the periodic box. The pressure was set to 1 atm and the temperature to 300 K, while using a time step of 2 fs, a non-bonded cut-off of 9 Å, rigid bonds, and particle-mesh Ewald long-range electrostatics. During minimization and NPT equilibration, the C_α_ atoms of residues from 12 to 32 are restrained by a 1 kcal mol^−1^ Å^−2^ spring constant. Finally, the production run is performed using ACEMD (31) on a NVIDIA Kepler K40 GPU for 200 ns. In addition, each system is simulated for 100 ns at temperatures of 350, 375, 400, and 450 K to test the temperature-dependent stability of the three different conformations. All the analyses are conducted after r.m.s. deviation convergence.

**Table 1:**
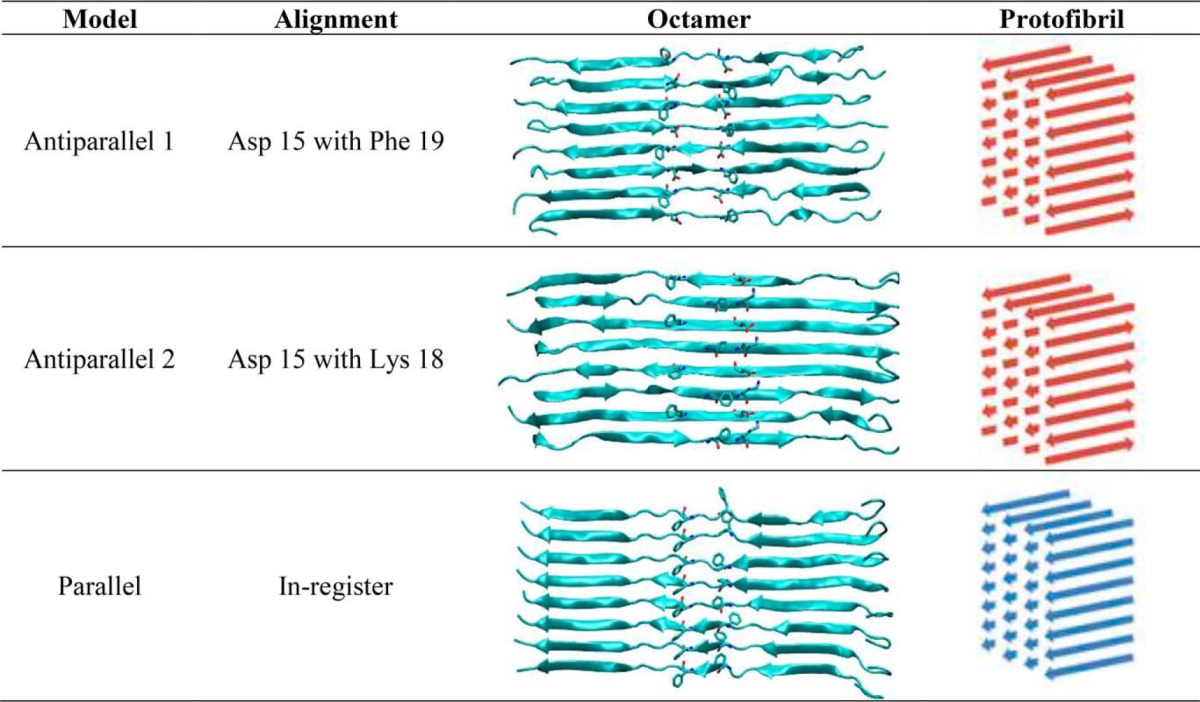
**Molecular models of calcitonin amyloid fibrils** Three different calcitonin octamers are employed to test the likelihood of three different proposed amyloid structures (antiparallel 1, antiparallel 2, parallel). The octamers are then assembled in protofibrils, to tests the influence of lateral interactions.

To test the influence of lateral interactions, we built three different protofibrils, one for each octamer conformation (Table 1). The protofibril models were obtained by duplicating the octamers and translating them by 10 Å along the *z* axis. Hence, each protofibril includes four octamers for a total of 32 hCT molecules. To test the stability of the different amyloid conformations, we performed 100-ns long classical MD simulations at the stable temperature of 300 K. In addition, we performed simulated annealing MD simulations in which the temperature increases from 300 to 500 K (5 K every 0.5 ns), then the systems are cooled down at the same rate and finally simulated at 300 K for 100 ns.

### DFNKF amyloid peptides

The initial structure of the DFNKF peptide is generated using PyMol (25). The initial assembly (octamers) in the form of parallel or antiparallel β-sheets is performed in VMD (32). The single peptide is aligned along the *x* axis, then we make 7 copies of the monomer and displace the copies along the *y* axis by multiples of 5 Å. Larger assemblies (protofibrils) were obtained by duplicating the β-sheets and displacing them by 10 Å (and multiples) along Direction 2 and by 20 Å (and multiples) along Direction 3 (see Fig. 9 and Table 2). Distances are chosen to allow close packing without excessive atom clashing.

**Table 2:**
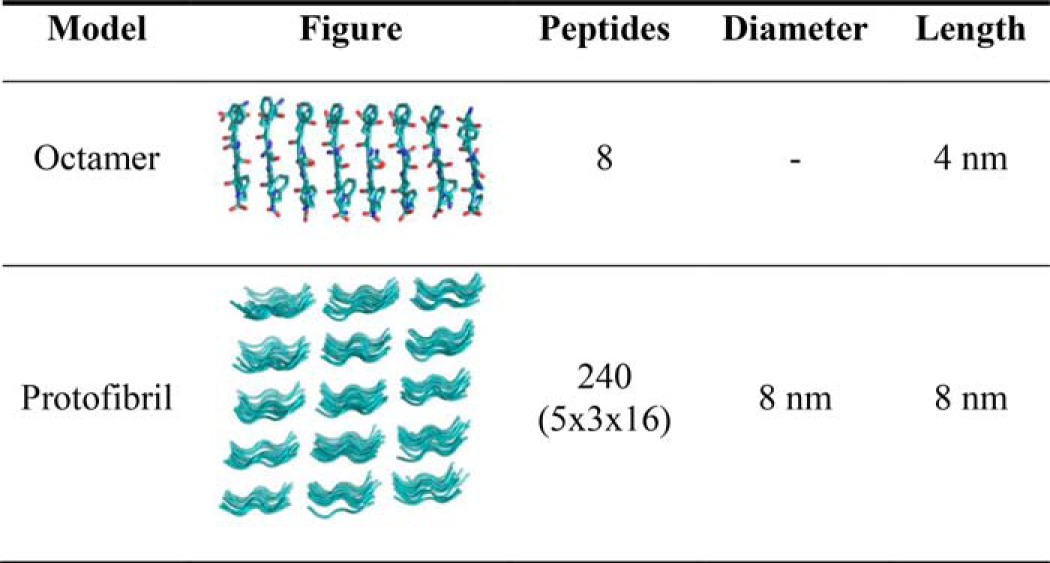
**Molecular models of DFNKF amyloid fibrils** Octamer models are used to test the preference for parallel or antiparallel nucleation. The full analysis of stabilizing interactions (longitudinal, lateral, and head-to-tail) is performed on protofibril model.

The octamers corresponds to assemblies similar to those studied in previous works (16, 37) and are meant to evaluate the stability of parallel and antiparallel configuration of the nucleation core. Protofibrils are investigated in all possible configurations using 72-mer protofibrils made of 8 × 3 × 3 DFNKF peptides. We thus investigated the 8 different protofibrils arising by combining the two possible configurations for each of the three directions (Fig. 9*B*).

The systems were solvated with TIP3P water molecules in a periodic box (with size depending on the amyloid dimensions). The systems were equilibrated using the same protocol described for the full-length calcitonin amyloid models. The stability (measured as β-sheets content) of the eight different assemblies was assessed through 100-ns long simulations at 300 K. For the most stable conformations we further investigate the stability by running simulations at increasing temperature up to 450 K. All analyses (*e.g.* hydrogen bonds, salt bridges, helix periodicity) are conducted using the VMD software package (32) and tcl scripting.

## Author contributions

P. M., A. R., and A. G. designed the research. F. R. and A. G. implemented the model and analysis tools, carried out the simulations, and collected the data. F. R., A. R., P. M., and A. G. analyzed the data and wrote the paper.

## Supplementary Material

Supplemental Data

## Footnotes

3 The abbreviations used are:

MD: molecular dynamics
hCT: human calcitonin
sCT: salmon calcitonin
REMD: replica exchange molecular dynamics
r.m.s.: root mean square
PDB: Protein Data Bank.
