# Supplementary material for: Nanostructure and stability of calcitonin amyloids
Source: J Biol Chem. 2017 Mar 10;292(18):7348–57. doi: 10.1074/jbc.M116.770271 (PMC5418037; doi:10.1074/jbc.M116.770271)
Supplement: Supplemental Data [file supp_292_18_7348__index.html]

Nanostructure and stability of calcitonin amyloids — Nanostructure and stability of calcitonin amyloids — Nanostructure and stability of calcitonin amyloids — Supplemental Data 

# Nanostructure and stability of calcitonin amyloids

## Supplemental Data

- Supplementari Figures (.docx, 323 KB) - Supplementari Figures
