# Supplementary material for: Nanostructure and stability of calcitonin amyloids
Source: J Biol Chem. 2017 Mar 10;292(18):7348–57. doi: 10.1074/jbc.M116.770271 (PMC5418037; doi:10.1074/jbc.M116.770271)
Supplement: Supplemental Data [file 10.1074_M116.770271_jbc.M116.770271-1.docx]

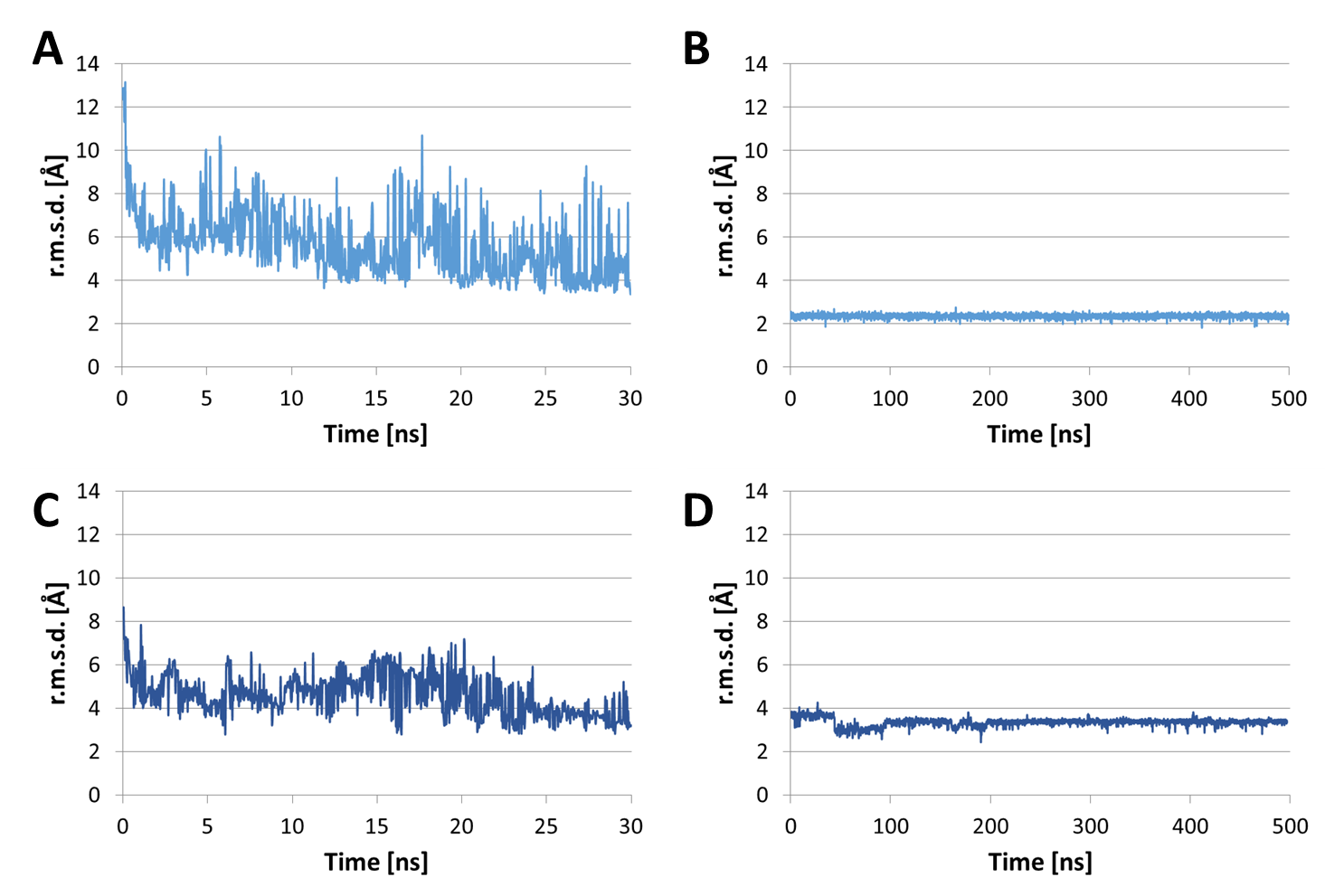


**Figure S1. R.m.s.d of sCT and hCT during REMD and MD simulations.** Panel (A) shows the r.m.s.d. trend during REMD simulation where the structural arrangement of salmon calcitonin moves from straight to helix conformation. Panel (B) shows high the stable r.m.s.d trend during classical MD simulation in explicit solvent. The r.m.s.d. trend for hCT during REMD simulations (C) and during classical MD simulation (C) show a behavior similar to that observed for sCT. All r.m.s.d. values are calculated restricting the analysis to backbone of residues from 4 to 21 and using as reference the NMR structure (PDB 2GLH).


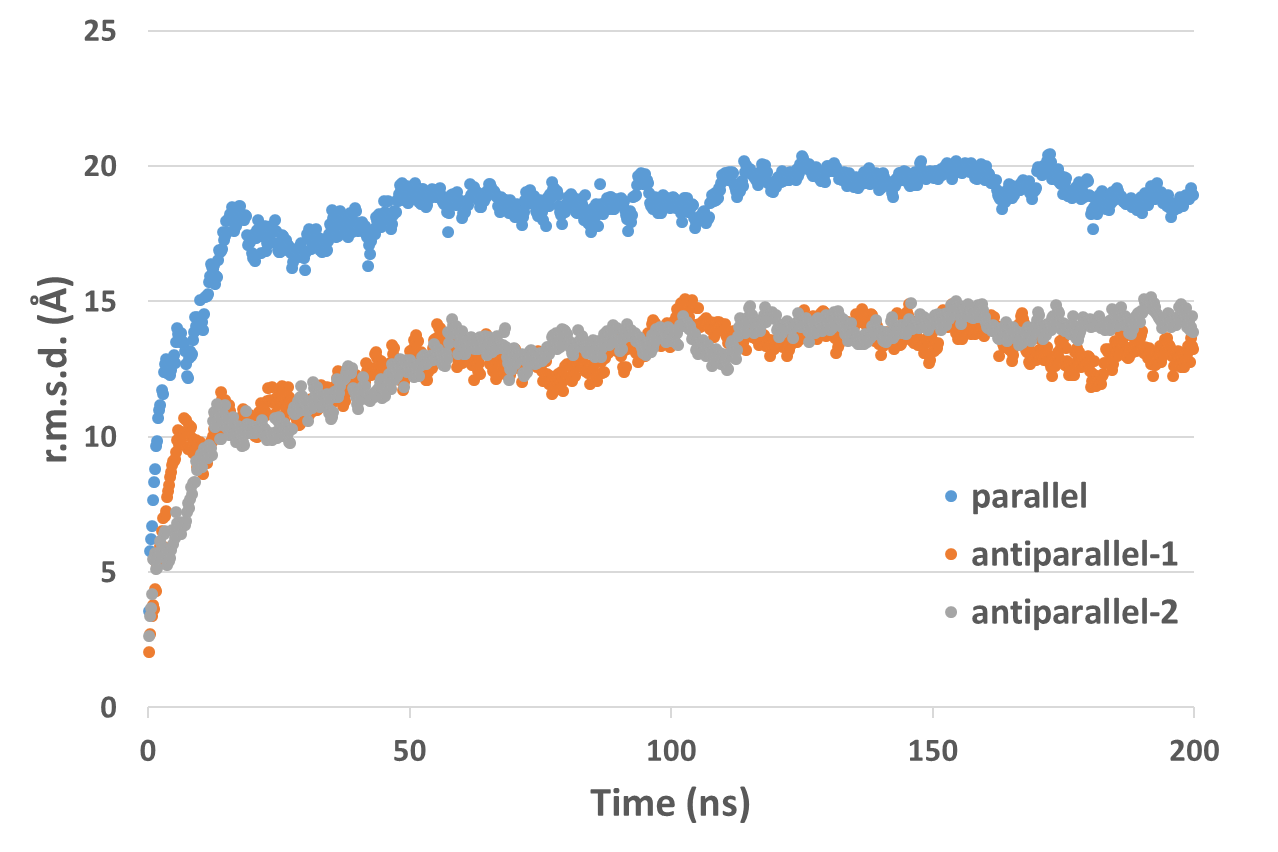


***Figure S2 – RMSD vs time for calcitonin octamers.*** *The trend of r.m.s.d vs. time during MD simulation of the three possible calcitonin octamer. As it can be observed, the r.m.s.d reached a stable asintotic value, meaning that the simulation has reached convergence.*
